# Mission and money: A scoping review of social enterprise business models as a solution to sustaining health-promoting food and nutrition-based initiatives

**DOI:** 10.1186/s12889-025-24891-7

**Published:** 2025-10-31

**Authors:** Leila I. Fathi, Jacqueline Walker, Clare Dix, Josephine Previte, Dilara Olgacher, Jayne Woodside, Helen Truby

**Affiliations:** 1 School of Human Movement and Nutrition Sciences, The University of Queensland, Human Movement Studies Building, St Lucia, Brisbane, QLD 4067 Australia; 2 Health and Wellbeing Queensland, 139 Coronation Dr, Milton, Brisbane, QLD 4064 Australia; 3 School of Health, University of the Sunshine Coast, Petrie, Moreton Bay, QLD 4502 Australia; 4 Business School, The University of Queensland, Colin Clark, 39 Blair Dr, St Lucia, Brisbane, QLD 4067 Australia; 5 Centre for Public Health, Institute of Clinical Sciences A, Queen’s University Belfast, Grosvenor Road, Belfast, Northern Ireland BT12 6BJ United Kingdom

**Keywords:** Social enterprise, Health promotion, Public health nutrition, Community, Entrepreneurship, Food, Nutrition

## Abstract

**Background:**

Most food and nutrition-based interventions are abandoned within two years of implementation due to a lack of secure long-term funding and political volatilities. Social enterprise structures are a potential solution to this problem due to their ability to generate profit and impact social outcomes. Therefore, this scoping review sought to explore what social enterprise models exist that address food and nutrition-based outcomes, and the factors that contribute to their long-term financial and operational sustainability.

**Methods:**

Scoping review methodology was utilised, with cross-checking currency of the social enterprise via Google (July, 2024). Eight search engines were explored, generating 2502 publications. Twenty-eight (20 case studies, 1 cross-sectional study, 6 evaluations and 1 quasi-experimental) studies were entered into a narrative synthesis.

**Results:**

Eight different operational models from 13 countries were identified: social cooperative; employment; targeted customer service; beneficiary service; market intermediary; social partnership; trading business; and social business. The most utilised model was the targeted customer service. The average duration of the enterprises was 16.5 years, showcasing that sustainable models that continue to deliver on social impact can have longevity.

**Conclusions:**

Social enterprises that adopt a business model from the outset are capable of generating income, whilst offering solutions to delivering on and sustaining food or nutrition-based outcomes within communities. There are several business models, though different, that have proven to have longevity. These models enable profit and social purpose to co-exist, benefiting the communities they serve.

**Supplementary Information:**

The online version contains supplementary material available at 10.1186/s12889-025-24891-7.

## Introduction

Globally, health promotion programs often rely on start-up funding from government and/or research grants. The long-term sustainability of programs requires consistent funding support, yet traditional financial institutions can prove unreliable past the point of efficacy testing. The issue of translating research or initiating programs into sustainable practice models points to the need to explore different approaches to generating income, so that health promotion programs can become financially self-sufficient and achieve their purpose over longer periods of time [1]. Despite showing early effectiveness, many health promotion programs cease after the period of initial funding, and therefore, fall short of being able to deliver measurable, long term and sustainable health benefits to their target audience [2]. School-based nutrition programs are an important example due to their ability to reach large audiences and address health behaviours that may track into adulthood but are often reliant on time-delineated government funding.

Our recent review explored the enablers and barriers that influenced the long-term (≥ 2 years) implementation of school-based nutrition programs [3]. A frequent barrier was a lack of organisational readiness and financial resources and a common enabler of success was having adequate external partnerships within a supportive environment [3]. The establishment and maintenance of robust relationships with stakeholders, alongside funding bodies, were crucial factors for health promotion programs that survived longer than 2 years [3]. Additionally, a qualitative exploration reporting on international school nutrition programs, revealed that the cyclical nature of external funding was a substantial barrier to the longevity of school-based nutrition programs [4]. To maximise the benefit of school-based nutrition programs and other, broader health promotion programs, alternative models of financial sustainability are required.

Social enterprises are a unique force with multiple innovation capabilities and have emerged in response to market and government failures to address wicked problems and meet diverse social, environmental, health and medical needs. The essential function of social enterprises is to merge the pursuit of public benefit and societal goals with the market-aligned tools and techniques of for-profit organisations [5]. Social enterprises therefore take many forms and span diverse sectors and are formed in response to social objectives, institutional factors and the resources at hand to tackle the problem [6]. 

At their core, is the pursuit of two types of value creation – commercial and social – integrating functions of commercial and social welfare logic. Macassa (2021) defines social enterprises as, ‘sustainable ventures that combine business principles with a passion for social impact’ [7]. This definition identifies an approach that differs from traditional philanthropic models where funding is donated to a specific cause via a charitable organisation. Financial support through donations or government funding is often unpredictable and insufficient [8]. As a result, the social enterprise business model has become established in many developed countries. These are increasingly a part of New Public Governance [9] structures that leverage involvement and engagement of societal actors in policy and service delivery in response to societal challenges through: (1) development or adoption of a solution (i.e. intervention/innovation); (2) ensuring the solution is accessible (i.e. intervention/innovation is scaled-up) and underpinned by an appropriate business model [10]. 

Multiple types of business models have been applied to support entrepreneurial strategies and they highlight how a for-profit logic of business aligns with identifying revenue-generating customers and the servicing of beneficiaries (i.e., the individuals and groups for who the social value is created). This includes the planning and methods of cost saving, generating new forms of revenue, resource conservation, long-term sustainability and effectiveness. Social enterprises deliver on social impact either directly, whereby the ‘intervention’ is the trading activity, or indirectly through a commercial activity that generates profits which can then be invested into assets [11]. What distinguishes social enterprises is the need to consider the *joint pursuit* of social and commercial value creation, and strategic endeavours that deliver social impact.

There have been successful examples of utilising social enterprise structures globally to improve social and environmental causes as described [12]. In 2002, the UK Government Department of Trade and Industry released a strategy, ‘Social enterprise: a strategy for success’, which aimed to strengthen the UK’s social enterprise sector, with a specific focus on health. There were approximately 70,000 social enterprises operating within the United Kingdom with 8–9% of the total providing National Health Service (NHS) funded care to the public [12]. In 2010, the UK Secretary of State for Health announced the ‘Equity and Excellence: Liberating the NHS’ strategy of the coalition Government which sought to further develop the social enterprise sector and increase the freedoms of NHS foundation Trusts and giving NHS staff greater autonomy in how income may be generated to support the health of the local population [13]. In 2011, approximately 10% of community health services had either launched a social enterprise or were in the process of finalising their business plans. Interestingly, in Australia, social enterprises are increasingly being established for administering the National Disability Insurance Scheme in an effort to control fiscal spending through the federal budget [12]. Overwhelmingly, social enterprises in both the UK and Australia are more likely to provide services (rather than the sale of products) in healthcare and social assistant sectors, and arts and recreation [14]. Despite promising examples of the use of social enterprise models to meet the health needs of communities, there remains a gap in our understanding of what business models may be applied effectively in the health promotion sector.

Chia et al. (2022) conducted a scoping review of social enterprises that addressed obesity or some of its component parts including physical activity, health literacy, access to healthy food and nutrition [15]. It identified 512 social enterprises in 32 countries, with almost half having ‘double-duty or triple-duty actions’, addressing obesity and other social or environmental determinants. However, it did not capture the breadth of social enterprises pertaining to food and nutrition-based missions and outcomes, such as food insecurity and food accessibility, and did not ascertain whether the duration of operation of the various enterprises.

This review aimed to elucidate what social enterprise models exist internationally that specifically address food and nutrition outcomes, and to elucidate the factors that contribute to their financial and operational sustainability to identify key business models or characteristics of successful long-term social enterprise models.

## Methods

### Search strategy

This review was guided by the 2020 Preferred Reporting Items for Systematic Review and Meta-Analyses (PRISMA) reporting guidelines and was not registered. The search for relevant articles was conducted on the 15th November, 2023, across eight business, social and biomedical databases: PubMed, Web of Science, Embase, Cinahl, Social Science Database, Sociological Abstracts, Business Source Complete, ABI/Inform Collection. No date or location parameters were applied. The search terms were initially developed for PubMed, consisting of the following terms: ‘social enterprise’, ‘social business’, ‘social venture’, ‘microenterprise’, ‘social entrepreneur’, ‘mission drive’, ‘food’, ‘nutrition’, ‘diet/food/nutrition’ (MeSH), ‘food assistance’ (MeSH), ‘food supply’ (MeSH), ‘diet’, ‘nutrient’. The terms were modified to suit all remaining databases with the assistance of the SR-Accelerator polyglot and the University librarian. The search terms and selected databases were deliberately broad to capture the breadth of the application of social enterprises that spanned health, social sciences and business. See Supplementary Material 1 for the search terms.

### Eligibility criteria

The definition from Chia et al. [15] was used to define a social enterprise as having ‘at least one revenue or resource stream that partly or entirely sustained its work and did not solely or mostly provide free products or services, whilst having a socially driven mission’. For articles to be included, they had to meet this definition, had to describe a food, water or nutrition-based social enterprise/s, that had an individual or combined focus on improving food, water or nutrition outcomes for people, communities or populations (for example, they had a focus on health promotion, disease prevention or treatment, or promoted accessibility to food or clean water for children or adults).

Articles were excluded if it could not be verified that the enterprise did not have a specific social purpose or mission, focused solely on food safety, only created employment opportunities (without having a food or nutrition-based mission or addressing food or nutrition based outcomes) or was involved in street vending, involved a sole-trader structure, involved micro-loans or loans of agricultural animals as the main business structure. Non-peer reviewed literature (such as grey literature or company reports), and non-English language articles were also excluded.

### Data handling

Retrieved articles were exported to Covidence [16] where duplicates were removed. Five researchers (LF, HT, JW, CD and DO) performed the title/abstract screening. The full text papers were screened by four researchers (LF, HT, JW and CD). Any conflicts were resolved by discussion (LF and HT).

### Data extraction

Study characteristics were extracted and transferred into an Excel spreadsheet by LF, which included information that pertained to the social enterprise mission and purpose, operational structure, legal status, the financial model (including income generation details), details of investors, product or service (see Tables 1 and 2). Grey literature and Google searches were conducted to determine whether the social enterprise was still operating in July 2024, which was used to determine the number of years of implementation. If there was no current operational web or social media presence for the enterprise, the years of implementation were determined by the peer-reviewed literature only, and if not reported or verified, was listed as ‘not reported’ (NR) see Table 2.

**Table 1:** Characteristics of included articles and descriptions of the social enterprises captured, including enterprise title, mission, the product/service/main activity and country of implementation

Study title	Year published	Study design	Enterprise title	Enterprise mission	Food or nutrition-based enterprise product/service/main activities	Country of implementation
Rebuilding child health in South Kivu, Democratic Republic of Congo (DRC): evaluating the Asili social enterprise program [17]	2022	Evaluation	Asili	To improve child health, reduce poverty and improve child survival through providing essential community services in South Kivu Province.	Clean water services	Democratic Republic of Congo
Improving Iodine Intake in Rural Haiti through Social Enterprise: A Cross-Sectional Study in the Central Plateau [18]	2023	Cross-sectional study	Bon Sel	To improve iodine intake in Haiti.	Production of iodised salt.	Haiti
Towards urban food sovereignty: the trials and tribulations of community-based aquaponics enterprises in Milwaukee and Melbourne [19]	2016	Cross-sectional comparative case study	Centre for Education and Research in Environmental Strategies (CERES)	To diversify local food production and improve community food security.	Urban aquaponics	Australia
How can social enterprises impact health and well-being? [20]	2018	Case study	City East	To improve community and individual health and wellbeing.	Food cooperative	Scotland
How can social enterprises impact health and well-being? [20]	2018	Case study	City North	To improve community and individual health and wellbeing.	Food cooperative	Scotland
Successful Niche Building by Social Innovation in Social Economy Networks and the Potential for Societal Transformation [21]	2024	Case study	Cretamo	To provide quality local products from small producers, enterprises and cooperatives with a low environmental footprint.	Food cooperative	Greece
Impact of the eKutir ICT-enabled social enterprise and its distributed microentrepreneur strategy on fruit and vegetable consumption: A quasi-experimental study in rural and urban communities in Odisha, India [22]	2020	Quasi-experimental study	eKutir	To provide self-sustaining solutions to poverty and undernutrition in developing countries by using information and communication technology.	Information and communication technology enabled ecosystem	India
The Intersection of Social and Economic Value Creation in Social Entrepreneurship: A Comparative Case Study of Food Hubs [23]	2019	Case study	Food Hub A (deidentified)	To improve access to healthy food.	Food hub - community garden organisation	United States of America
The Intersection of Social and Economic Value Creation in Social Entrepreneurship: A Comparative Case Study of Food Hubs [23]	2019	Case study	Food Hub C (deidentified)	To improve access to healthy food.	Food hub	United States of America
Social enterprise, sustainability and community in post-earthquake Christchurch: Exploring the role of local food systems in building resilience [24]	2017	Quasi-case study	Garden City 2.0	To support the growth of the local organic food sector and enhance the food growing capacity of greater Christchurch in a self-sufficient manner.	Supply of local organic food	New Zealand
Business-Based Strategies for Improved Nutrition: The Case of Grameen Danone Foods [25]	2018	Case study	Grameen Danone Foods Ltd	To improve the micronutrient status of poor children in Bangladesh.	Sale of a fortified yoghurt (Shokti+)	Bangladesh
Grameen Danone Foods - A Case of a Social Business Enterprise [26]	2011	Case study	Grameen Danone Foods Ltd	To reduce poverty by providing daily healthy nutrition to the poor through a proximity model.	Sale of a fortified yoghurt (Shokti+)	Bangladesh
Grameen Danone Foods Limited (GDF) [27]	2012	Case study	Grameen Danone Foods Ltd	To provide affordable, good nutrition to Bangladeshi children.	Sale of a fortified yoghurt (Shokti+)	Bangladesh
Social business and Grameen Danone Foods Limited [28]	2013	Case study	Grameen Danone Foods Ltd	To reduce poverty through a proximity model that provides good nutrition to the community.	Sale of a fortified yoghurt (Shokti+)	Bangladesh
Social Responsibility, Business Strategy and Development: The Case of Grameen-Danone Foods Limited [29]	2009	Case study	Grameen Danone Foods Ltd	To create local employment opportunities and provide affordable nutrition to children.	Sale of a fortified yoghurt (Shokti+)	Bangladesh
A mixed-methods evaluation of community-based healthy kitchens as social enterprises for refugee women [30]	2019	Mixed-methods evaluation	Healthy Kitchens, Healthy Children (HKHC) intervention	To alleviate long-term food insecurity in two Palestinian refugee camps in Beirut, Lebanon.	Healthy snacks	Lebanon
Hidden Harvest’s transformative potential: An example of ‘community economy’ [31]	2018	Case study	Hidden Harvest Ottawa	To rescue urban produce (fruit and nuts that would otherwise go to waste) and share it with those in need.	Food redistribution	United States of America
Growing in Glasgow: Innovative practices and emerging policy pathways for urban agriculture [32]	2017	Embedded case study	Locavore	To provide consumers with local and sustainably produced foods as an alternative to dominant supermarket chains.	Urban agriculture and produce delivery and shop	Scotland
Between a business and a social enterprise The Norway House Fisherman’s Co-op, northern Manitoba, Canada [33]	2016	Mixed-methods evaluation	Norway House Fisherman’s Co-op	To increase local food security and contribute to the economic development of the local community.	Food cooperative	Canada
Social enterprise, sustainability and community in post-earthquake Christchurch: Exploring the role of local food systems in building resilience [24]	2017	Quasi-case study	Shop Eight Restaurant	To make local food interesting and accessible.	Provision of local food	New Zealand
Social-ecological systems and social innovations regarding fruits and vegetables supply chains – case study of the Brazilian social enterprise Sumá [34]	2022	Case study	Sumá	To promote sustainable food systems.	Network of farmers and food purchasers	Brazil
Towards urban food sovereignty: the trials and tribulations of community-based aquaponics enterprises in Milwaukee and Melbourne [19]	2017	Cross-sectional comparative case study	Sweet Water Organics (SWO)	To develop capacity, employment opportunities, and fresh and affordable food for the local community.	Urban aquaponics	United Sates of America
The impact of social enterprise on food insecurity - An Australian case study [35]	2019	Mixed-methods evaluation	The Community Grocer	To improve food security.	Fruit and vegetable market	Australia
The Empty Bowls Project: Creating, Leading, and Sustaining a Social Enterprise: ET&P [36]	2013	Case study	The Empty Bowls Project	To improve food security and to fight hunger.	Fundraising event, providing a handcrafted bowl and soup	United States of America
Food-Based Social Enterprises and Asylum Seekers: The Food Justice Truck [37]	2018	Case study	The Food Justice Truck	To provide affordable, nutritious food to people seeking asylum, and alleviate food insecurity.	Mobile fresh food market	Australia
The role of social enterprise in food insecurity among asylum seekers [38]	2018	Mixed-methods evaluation	The Food Justice Truck	To provide fresh produce that is subsidised for asylum seekers vulnerable to food insecurity.	Mobile fresh food market	Australia
Fighting Hunger Through Innovation: Evaluation of a Food Bank’s Social Enterprise Venture [39]	2010	Case study	The Market	To improve the accessibility, availability and affordability of food.	Agency run grocery store	United States of America
Accidental expert: Experiments in sustainable restaurants and food retailing [40]	2011	Case study	The People’s Supermarket	To make sustainable food more accessible and to create better local food distribution.	Food cooperative	England
Social Entrepreneurship and Social Movement Learning: A Reflective Account of the History of the TPSS Food Cooperative [41]	2019	Case study	TPSS Food Co-Op	To provide healthy food to the community.	Food cooperative	United States of America
Beyond Charity? Insights on the Upcycle Kitchen: A Food Rescue Work Integration Social Enterprise in Guelph, On [42]	2023	Case study	Upcycle Kitchen	To improve food security and reduce food waste.	Food rescue work integration and provision of food	Canada
Growing in Glasgow: Innovative practices and emerging policy pathways for urban agriculture [32]	2017	Embedded case study	Urban Roots	To improve the accessibility of fresh food in the community and provide employment opportunities for local people.	Urban agriculture	Scotland
Vikings Table: An Innovative Social Venture [43]	2020	Case study	Vikings Table	To advance the wellbeing of youth through engaging health and education initiatives.	Food truck	United States of America
A mixed-methods evaluation of a health-promoting café located in a small health service in rural Victoria, Australia [44]	2022	Mixed-methods evaluation study	YarriYak Café	To provide healthier food and to increase employment opportunities for clients of the local disability service.	Café	Australia

**Table 2:** The type and description of the social enterprises' operational models, legal status, financial model, stakeholders involved and years of operation

Social enterprise	Operational model of social enterprise	Description of operational model	Legal status	Financial model	Stakeholders involved	Years of operation
Asili [17]	Social cooperative model Targeted customer service model Beneficiary service model	• Provides access to health services, clean water and an agricultural cooperative through a membership-based model. • Asili members receive cans of water per day and subsidised prices for clinic appointments and medicine. • Franchisable model with opportunities for job creation. • Delivered through local partners: (1) clean water kiosks developed by Associations des Usagers de Reseaux d’Eau Potable; (2) small format health clinics (based on the Kenyan and Rwandan HealthStore model); and (3) agriculture co-operatives encouraging improved agricultural practices including production, use of seed varieties, and household garden (based on the Action Sociale d’Organisation Paysanne cooperative model).	NR	• Community members could access Asili either through paying a monthly membership or purchasing services individually. Asili users (not members) pay higher fees for cans of water, clinic services and medicine.	• The co-creators were the American Refugee. Committee and Ideo.org • Supported by funding from USAID, private donors and social investors. • Incubated by Alight.	10*
Bon Sel [18]	Market intermediary model	• Bon Sel is Haiti’s exclusive iodised salt supplier, operating a commercial processing factory. • Small-scale producers play a key role by selling unprocessed salt (making a strong market contribution) to a local processor that handles purification, fortification and packaging. • The salt is sold under the Bon Sel Dayiti brand name.	Not for profit	• Sale of iodised salt products occurred in three formats: processed foods (bouillon, bread), food service (school meal programs), and retail (packaged sachets).	• The Congregation de St. Croix, Haiti (within the University of Notre Dame US Global Center for the Development of the Whole Child) owned and operated the salt processing factory. • Partnered with the Haitian Ministry of Health.	18*
Centre for Education and Research in Environmental Strategies (CERES) [19]	Trading business model	• CERES is a centre for education and research, focusing on environmental strategies. • It operates a commercial, urban aquaponics system. • The produce is sold to the CERES Fair Food organic box delivery enterprise that is located in a nearby facility.	Not for profit	• Initial grant funding. • Sale of produce directly to the CERES Fair Food organic box delivery enterprise. • It has the production capacity to support the wage of a single farmer (reimbursed through the sale of produce).	• The CERES Green Technology team manager was Steve Mushin.	14*
City East [20]	Social cooperative model	• It grew from a federation of food co-operatives and targets deprived communities in Scotland. • There are 15 full-time employees (inclusive of six drivers) and seven part-time staff members.	NR	• Conducts trading. • Many staff are funded by community-based job creation schemes.	NR	25
City North [20]	Social cooperative model	• It operates a hub and spoke business model (with three food hubs). • Targets asylum seekers and the local community living in income and employment deprivation and children living in poverty. • Operated by four full-time employees, six part-time staff (including one driver, one nutritionist), one gardener and a volunteer co-ordinator. • 40–50 volunteers are recruited and supported on an annual basis.	Not for profit	• Conducts trading.	• In 2001, it was initiated by Glasgow University students in response to an increase of asylum seekers being located to north Glasgow.	23*
Cretamo [21]	Social cooperative model	• A social cooperative operating a grocery store in Thessaloniki. • Operates by paid and volunteer work. • There are more than 430 members and is open to new members (anyone can join). • The store serves everyone, including non-members.	Not for profit	• Capital was generated by contributions from its initial members (members bought a share for 150 euros). The capital was used for rental cost and obtaining equipment and supplies. • Creatmo is self-supported through members’ contributions and grocery store revenue. • Profits are shared among the cooperative members in the form of rebates (certain discounts on the transactions).	• Cretamo has committees for product promotion, selection and collaborations with other initiatives.	8
eKutir [22]	Social partnership model	• eKutir leverages a digital ecosystem supported by an information and communication technology platform to empower farming micro-entrepreneurs. • Local and regional NGOs, agricultural input organisations (such as seed, fertiliser companies), agricultural experts, cart manufacturers, cold storage suppliers, rural banks, and other actors in the system, can access the ecosystem. • This ecosystem promotes demand by the consumer, improves productivity and efficiency of the value chain on the supply side, and supports low-resourced communities. • Farming micro-entrepreneurs are trained by the social enterprise to use mobile software applications to provide inputs, technical assistance, market linkages, and daily market pricing information for smallholder farmers (participating in the eKutir program). • Farmers are grouped into ‘Farmer Intervention Groups’ (15–25 members) by the farming micro-entrepreneurs, whose produce is aggregated by farmer medium enterprises and funnelled into the VeggieKart distribution channel. • After processing (weighing, sorting, grading, packaging), the vegetables are distributed through eCommerce or through retail channels which consist of: VeggieMart (small shops in farmer’s markets), VeggieWheels (vendors with push karts) or VeggieLite (affordable fresh produce for low-income customers in a retail setting).	For profit; certified B-corp	• Sale of fresh produce. • Funding was received from government and various foundations.	NR	15*
Food Hub A (deidentified) [23]	Market intermediary model	• Operates as a food hub, with a marketing channel.	Not for profit	• Revenue is generated by charging supplier/producer fees for using the hub. • Funding was received from a foundation, non-profit organisations, local community foundation, and federal government programs.	NR	NR
Food Hub C (deidentified) [23]	Market intermediary model	• Food hub structure of operation. • The food hub is used as a marketing channel. • It is a project of a larger non-government organisation. • Aimed at local farmers and food processors.	NR	• Funding was received from a non-profit organisation.	NR	NR
Garden City 2.0 [24]	Targeted customer service model	• Combines community work, education on nutrition and growing food (to promote community resilience), and an organic food delivery service. • A weekly produce shop operates in Summer. • Uses its online presence (via website and social media) for community outreach and engagement to promote services.	NR	• Sale from its food bag delivery service and produce shop.	• The Chair of Soil and Health Canterbury.	NR
Grameen Danone Foods Ltd [25–29]	Targeted customer service model Beneficiary service model	• Manufactures and distributes two products: (1) a fortified yoghurt called Shokti+ (requires refrigeration), and (2) Shokti Pocket (does not require refrigeration). • There are four distribution channels: (1) door-to-door sales through local women (bought through microcredit and sell with profit); (2) sales through small retail shops in a rural area; (3) urban distribution through retail shops; (4) distribution to high-income markets and high-end grocery stores. • When the product is sold at a premium to high income markets and grocery stores, it is sold in distinct packaging from the other sales routes. • Adopted a close-proximity business model (suppliers and consumers in close-proximity to the manufacturing facility). • The milk (primary ingredient) is sourced from local dairy farmers who are able to acquire the cows through micro-loans from from Grameen Bank.	Not for profit	• Groupe Danone provided US$1 million to construct a milk processing factory. • Funded by Groupe Danone through its 4 subsidiaries. • Shokti + sales for 10BDT (US$0.12). • Shokti Pocket sales for 6BDT (US$0.07). • Shokti + for high income markets sales for 30BDT (US$0.37).	• Established as a joint venture between the French food conglomerate Groupe Danone (a global manufacturer and distributor of yoghurt) and Grameen Enterprises/Grameen Bank of Bangladesh (a major NGO engaged with poor populations in Bangladesh). • The Global Alliance for Improved Nutrition helped establish the initial formulation of Shokti+.	18*
Healthy Kitchens, Healthy Children (HKHC) intervention [30]	Employment model	• Two community kitchens (small business enterprises) are run by Palestinian women who provide healthy snacks to Palestinian refugee children in UN Relief and Works Agency (UNRWA) schools, for one academic year. • The intervention involved a week-long training for the women, covering topics like entrepreneurship, food preparation, food safety, nutrition and the development of standardised recipes of healthy school snacks. • The women with a nutritionist worked on creating a monthly snack menu for the primary school aged children. • Women would then cater healthy school snacks to children daily who were attending the two UNRWA schools. • At the time of publication, the intervention developed into a sustainable catering business, providing food for schools, a pre-school, an orphanage and local events.	NR	• Revenue is from subsidised snack sales to school children (0.25USD per snack or 5USD per month for 20 snacks). • The women earned 110USD per month from snack sales and subsidy.	• Funding was provided by the Nestle Foundation for the Study of Problems of Nutrition in the World. • Support was provided by the UN Relief and Works Agency for Palestine refugees and existing community-based women’s organisations.	0.7
Hidden Harvest Ottawa [31]	Social business model	• Using city data that revealed the location of food-bearing trees on city property, and mapped data by Hidden Harvest groups, groups of volunteers participate in harvest events, organised by ‘neighbourhood leaders’ who are trained by Hidden Harvest. • The food collected is distributed as follows: 1/4 for a local food agency (shelter or food bank), the remaining 3/4 is divided equally among the volunteer, harvesters, Hidden Harvest, and the homeowner (if fruit came from a private property).	For profit	• Received grant funding. No one was paid unless grants were received. • Hidden Harvest raise funds for the initiative by selling their share to local restaurants and processors. • Hidden Harvest monetises their ¼ share of fruit by receiving a share of profits of the final product made by local food processors (products including preserves and beer). • In 2012 it attempted to raise funds through the sale of food-bearing trees but it did not generate sufficient profit to support core organisational operating costs. • Hidden Harvest partnered with (1) Bridgehead Coffeehouse to receive revenue from sales on a fundraiser coffee; and (2) Beau’s All Natural Brewing Company.	• Partnered with Oak Computing to develop a website to allow the public to register trees for harvest and sign up to volunteer. • For future and potential harvests, an interactive map and email notification system were created. • Hidden Harvest Ottawa had established a steering committee.	12*
Locavore [32]	Targeted customer service model Employment model	• Sell locally produced food through a delivery service. • The shop supplied a variety of locally produced vegetables to subscribers every week. • They provide work for unemployed young people to deliver the vegetable boxes on bicycles. • To meet demand, fruit and vegetables are bought from local community garden projects and from rural organic farms. • In 2014 a market garden site was established to meet demand and control supply (through ‘nano market gardening’) on vacant land. Interested gardeners grew crop for the vegetable box scheme and the shop provided educational advice to the growers.	Community interest company	• Established through a scheme associated with the Big Lottery Fund. • Crowd-funding was used to establish a social enterprise supermarket. • Revenue is generated through the sale of the vegetable boxes.	• Established by one of the original founders of South Seeds (another social enterprise).	12*
Norway House Fisherman’s Co-op [33]	Social cooperative model	• A membership-based cooperative where all commercial fishers must join to sell their catch, complying with Manitoba law that requires sales to be made to Freshwater Fish Marketing Corporation (possible through the cooperative). • Fishers deliver their catch to one of two seasonal processing stations, where the fish are then packaged and transported to a plant in Winnipeg for further processing and marketing.	NR	• Membership based model. • Yearly profits are invested in business diversification – the diversity of businesses provide employment and contribute to community economic development. • The co-op operates a lumber business, a convenience store, a petrol station, and a fast-food franchise (where they sell value-added fish products). During fishing season, the co-op sells fishing equipment. These services assist fishers and community members to get products and services at a reasonable cost.	NR	62*
Shop Eight Restaurant [24]	Targeted customer service model	• Began as a local food initiative offering pizza made with hyper-local ingredients that are sourced from local growers. • It is a small restaurant committed to sourcing locally and supporting food education through initiatives and events.	NR	• Revenue is generated from the sale of food.	• It was supported by the Christchurch City Council and Gap Filler.	NR
Sumá [34]	Social cooperative model Social partnership model	• Sumá trains and organises small farmers to form cooperatives and connects them to regular food purchasers in small towns, and acts as the intermediary of deals. • Food purchasers include public procurement programs and contracts (such as the National School Feeding Program-PNAE) and private purchasers (food service companies).	NR	• Various organisations have invested in Sumá, including four venture capital, angel investing or impact investing activities. • In the company’s financial structure, there are two sources of revenue for the intermediation of deals, search for buyers, and training. • Downstream in the chain, there is the sale of agricultural products with 20% of the value directed to Sumá. Of the remainder, 15% goes to the cooperative (which manages the assembly of the product), 15% to drivers, and 50% to the producer. • Corresponding to upstream activities, work is done with sponsorships to remunerate the qualification of farmers.	NR	NR
Sweet Water Organics (SWO) [19]	Social business model Trading business	• The urban aquaponics farm was set up in an unused, inner city, industrial building. • In 2010, it evolved into a hybrid organisation when Sweet Water Foundation was split from Sweet Water Organics. The new model includes a commercial urban farm (for-profit), and an aquaponics “academy” (not-for-profit). • Engages local community and schools to educate people how to grow food and address social issues.	For profit	• Funded by founders James Godsil and Josh Fraundorf • In 2011 $175,000 was won to develop an online digital training and credentialing platform. • A ‘forgiveable’ loan was granted from the city to upgrade its model. • In 2012 it was sustained through loans financing and equity investment. In 2013 they were not able to sustain loan repayments.	• The founders were James Godsil and Josh Fraundorf.	5 (ceased operation in 2013)
The Community Grocer [35]	Targeted customer service model	• Operated six concurrent weekly fruit and vegetable markets, managed by paid staff and volunteers. • Fresh produce were purchased weekly from a commercial wholesale business (delivered on market day).	Not for profit	• Revenue was generated from produce sales. • It was supported by philanthropic funding and volunteer work which enabled the purchase of equipment, materials and allows the market to operate at a low-cost (products priced at about 30% above wholesale).	• It was founded by two experienced not-for-profit food security practitioners. • The Board of Directors are responsible for the governance and financial sustainability.	10*
The Empty Bowls Project [36]	Social business model	• Potters, students and other interested individuals create handcrafted bowls which are donated for an Empty Bowls event. • At the event, a meal of soup and bread are provided in the bowls in exchange for cash donations. • The attendees take the bowl home as a symbol of global food insecurity. • Funds are donated to community-based organisations working towards food security. • It is recommended that a representative from the local agency receiving the donation present at the event about food insecurity. • Individuals from around the world can register and host their own event.	Not for profit	• Donations collected at events support community-based organisations working towards food security.	• The founders are Lisa Blackburn and John Harton.	34*
The Food Justice Truck [37, 38]	Targeted customer service model	• The Food Justice Truck (FJT) was a mobile subsidised fresh food market that operated on a weekly or fortnightly basis. • It provided ethically sourced and locally produced fresh vegetables, fruit, pulses, grains, bread, and tea to asylum seekers at a discounted price and also to the public at market rates.	NR	• Received community and philanthropic funding. • The FJT purchased foods from low-cost suppliers. • Pricing of the FJT food was calculated according to government payments that asylum seekers receive. • A 60%−75% discount for asylum seekers was offered (asylum seekers could purchase $80 worth of food for $20), whilst maintaining full prices for the general public.	• Established by the Asylum Seeker Resource Center which relies on community and philanthropic funding.	2.5 (ceased operation in 2017)
The Market [39]	Targeted customer service model	• The Food Bank of Delaware started a discounted store for food insecure/low-income people in Newark and Wilmington, Delaware. • Staffed by volunteers and employees of the food bank. • The store sold goods purchased from a salvage vendor (paid in advance for a truckload of product). The delivery of products varied and the food bank was not able to select the specific products. • Milk, bread and eggs were bought from a local merchant and became available to purchase, to increase the convenience of the store.	NR	• The Market had private donors. • Donated food or products were not sold at the market. • The Pilot Market began with a low cost (about $5000) to the food bank. Payment for rent or utilities was not required due to the store being located in the warehouse. • Merchandise was purchased from a salvage goods distributor and then sold either above cost or occasionally at cost to the customer. • Profit that was received went back into the food bank to purchase new stock or to their overall mission.	• Developed by the Food Bank of Delaware.	NR
The People’s Supermarket [40]	Targeted customer service model	• Membership based supermarket that involves members working for four hours a month and in return they receive 10% off at the store. • Everything in the shop has been reused or recycled (wall tiles, second-hand tills, reconditioned fridges).	Not for profit	• Received some help from local government. • Suppliers are carefully selected so that food can be sold at the supermarket at low prices.	NR	NR
TPSS Food Co-Op [41]	Social cooperative model	• People join with an initial financial investment and volunteer at the co-op. • Members and non-members can shop at the co-op. • They conducted educational outreach to the community about healthy vegetarian food and alternative sources of healthy food. • A community cookbook of recipes was created, and cooking classes were conducted. • A catering collective was sponsored that used products and recipes from the store to support events. • A vegetarian cafe that featured recipes and products from the co-op, hosted music nights and had speakers focusing on current social issues. • Once the enterprise grew and was located to a more central area in the community, the membership changed to be based on a membership fee which then provided a discount on food purchases.	Not for profit	• Revenue is generated from membership fees.	• It started with the board of directors making decisions but then moved to employing a general manager so that they could focus on policy. • Board of directors made decisions about what products to carry, volunteer involvement, and equipment.	43*
Upcycle Kitchen [42]	Employment model Targeted customer service model	• A work integration model that improves access to healthy food, employs out-of-work youth, and addresses food waste. • Surplus produce that would have been wasted is rescued, which is then processed or upcycled into high quality value-added products and meals, which are sold at the SEED’s market and an Upcycle Kitchen Café. • Once per week the Upcycle Kitchen Cafe serves prepared lunch to the Guelph Community Health Centre community based on a sliding cost scale to offset operational costs. • For the Upcycle Kitchen, most of the food supply is donated rather than purchased (such as from the SEED Community Food Markets).	Not for profit	• Donations came from external donors and surplus food from the SEEDS’s internal projects. • Had start-up grant funding from several foundations. • Consumers can purchase products at a low-cost using sliding scale pricing at The SEED’s Community Food Markets (where Upcycle Kitchen products are sold). Profit is reinvested back into the enterprise to offset operational, staffing and processing costs.	• Upcycle’s parent organisation is the SEED, a project of the Guelph Community Health Centre (a registered charity). • SEED was established through research done through a community-university partnership with the University of Guelph that identified that the existing food program to be inadequate in providing access to fresh food.	4*
Urban Roots [32]	Social business model Targeted customer service model	• Initially called Toryglen Gardening Club, established a number of community gardens. • In 2008 in became a registered charity and was renamed to Urban Roots. • Through community engagement activities, it helped various organisations to develop skills and capacities in garden design, construction and implementation. • In 2009 it signed a lease with Glasgow City Council to manage the Malls Mire Woodland. • It also conducted workshops for local community members on growing and cooking and hosted various harvest festivals. • In 2012, it took over a new site and planted a community market garden. The garden generated a modest income through selling a small amount of fresh produce in the local area.	Registered charity	• Revenue was generated through the sale of fresh produce. • Urban Roots wanted to generate income from Polmadie Plots by selling produce to local shops and social enterprises. They also generated income through offering consultancy services to larger organisations with which it worked. • Funding was obtained from the Fair Share Trust for a permanent staff to be hired, the National Lottery’s Fair Share Trust, the Scottish Government’s Climate Challenge Fund and the Scottish Government (as urban agriculture became a popular national policy objective). • In 2014, Urban Roots received support from the Scottish Government’s People in Communities fund, the Central Scotland Green Network, the Heritage Lottery Fund and via the NHS’ Community Health Partnerships.	• Established by local residents who shared a passion for community activism and sustainability.	19*
Vikings Table [43]	Social business model	• A custom made food truck which is present at all Vikings events and sells food to patrons of those events. The proceeds fund the meals program and nutrition education for inner-city children.	NR	• Profits from the food truck were used to achieve the enterprise’s mission. • Funding was provided by the Minnesota Vikings National Football League team, Second Harvest Food Bank, individual and corporate donors.	• The lead stakeholders included The Minnesota Vikings National Football League team and Second Harvest Food Bank.	5*
YarriYak Café [44]	Employment model Targeted customer service model	• Through a whole community food environment audit, it was identified that there was poor accessibility healthy food in the area. This enhanced motivation to establish a health-promoting café. • The café was established and operated by Woodbine, a local disability service. Woodbine provides hospitality staff and products that are sold in the café. A traffic light system is used for the food products and provides healthy food options for the community members and employees of the Rural Northwest Health Service.	NR	• Revenue is generated from the sale of café products.	• Bernie O’Connor was the CEO of Woodbine.	8*

*NR* Not reported in the peer-reviewed literature or no grey literature to determine years of operation

* Still in operation in 2024

### Synthesis of results

Based on the descriptive factors of a social enterprise’s business structure, its operational model was determined. Table 3 synthesises the operational business models, including their key defining characteristics, the number of social enterprises that utilised each model and the social enterprises' average years of operation which was used to inform the business model’s sustainability.

**Table 3:** Summary of the social enterprises' operational models, including characteristics, number of social enterprises using the model and average years of implementation, noting that some enterprises operated a combination of models

Social enterprise operational model	Description of model characteristics	Number of social enterprises	Average years of implementation*
Social cooperative model	• Individuals join with an initial financial investment which may include renewal fees. • Members receive benefits from the social enterprise such as discounted products or services. • Individuals from the public who are not members of the cooperative may pay the premium for the cooperative’s products or services.	7 [17, 20, 21, 33, 34, 41]	29
Employment model	• Disadvantaged members of a community are given opportunities for employment and skill development e.g. individuals with disabilities. • The group of interest is determined by the enterprise.	4 [30, 32, 42, 44]	6
Targeted customer service model	• A social enterprise may offer a specific product, an array of products or services in exchange for a fee from the consumer or target population.	12 [17, 24–29, 32, 35, 37–40, 42, 44]	10
Beneficiary service model	• A form of fee-for-service that targets low-income individuals of a target population. • The low-income individuals are the market of interest; however, they also benefit from the product or services the social enterprise is offering.	2 [17, 25–29, 45]	14
Market intermediary model	• The social enterprise is a resource integrator in the value chain, whereby it purchases a product from small producers, add value to the product or assist in its marketing, and sells it to the target market at a profit.	3 [18, 23]	18
Social partnership model	• The social enterprise links producers of a product to a market, they otherwise would not have had access to. • The social enterprise generates revenue through connecting the producer to the market of interest. • The social enterprise plays no role in selling or marketing the producer’s product.	2 [22, 34]	15
Trading business model	• A social enterprise sells its products or services to a targeted market which then reaches beneficiaries of the social mission. • The revenue generated can help cover the cost of its social programs or outputs. • Its business activities are “mission-unrelated” in that earning income is separate from its social programs and focused on financial support for the social mission.	2 [19]	10
Social-business model	• A social enterprise sells products or services to a specified target population and the revenue generated is used to fund social programs that are aligned to its mission.	5 [19, 31, 32, 36, 43]	15

* Excludes 7 social enterprises that did not have data on years of implementation available

## Results

### Study selection

**Fig. 1:**
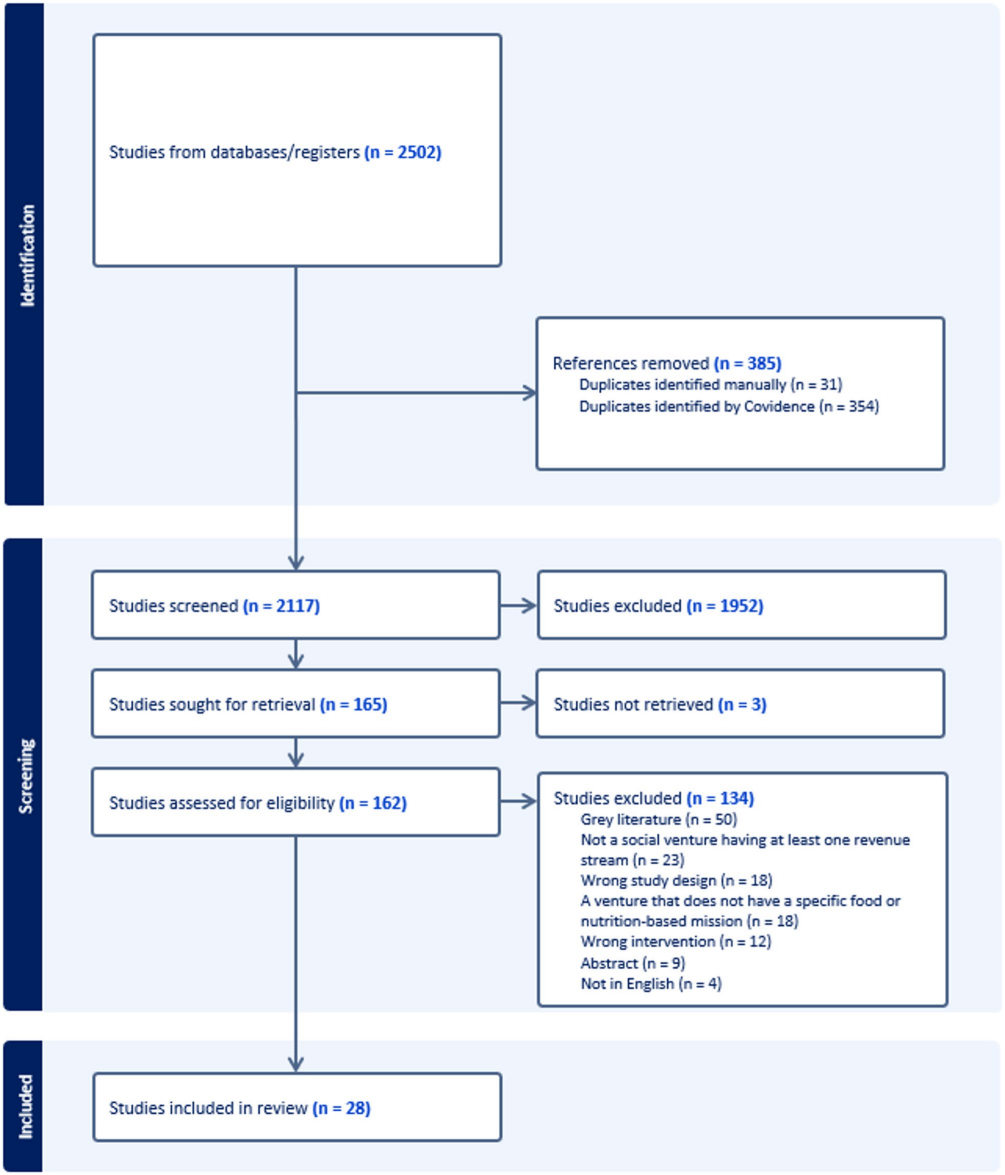
PRISMA flow chart for included and excluded articles

From the database searches, 2502 articles were identified, from which 385 duplicates were removed. The title and abstract screening involved 2117 articles from which 1952 were excluded. This resulted in 165 articles being eligible for the full-text screening, with 3 articles not able to be retrieved despite corresponding authors being contacted to request information. This resulted in 162 studies entering full text screening and, after 134 articles were excluded, a total of 28 articles were included in the review. Refer to Fig. 1 for the full PRISMA flowchart [46]. 

### Review outcomes

The 28 included articles consisted of 20 case studies, 1 cross-sectional study, 6 evaluations and 1 quasi-experimental study. Details of the business model of these different social enterprises were extracted and classified (done by LF and verified by JP) according to terminology from Grassl (2012) [47], De Cuyper et al. (2024) [48], Defourny et al. (2021) [49], and Gupta and Chauhan (2020) [5]. Any assumptions were cross-checked via a web-based search to verify specific details of their mission and purpose. The variation in study design plus the cross-disciplinary databases searched which ranged across health, social sciences and business, resulted in the papers reporting on a variety of outcomes, therefore, it was not appropriate to conduct a formal quality review of methodological rigour.

Twenty-eight social enterprises were identified that were located in 13 countries: Australia (*n* = 4) [19, 35, 37, 38, 44], Bangladesh (*n* = 1) [25–29], Brazil (*n* = 1) [34], Canada (*n* = 2) [33, 42], Democratic Republic of Congo (*n* = 1) [17], England (*n* = 1) [40], Greece (*n* = 1) [21], Haiti (*n* = 1) [18], India (*n* = 1) [22], Lebanon (*n* = 1) [30], New Zealand (*n* = 2) [24], Scotland (*n* = 4) [20, 32], and United States of America (*n* = 8) [19, 23, 31, 36, 39, 41, 43]. Almost all enterprises only operated in their country of origin, except for The Empty Bowls Project from the United States of America which had expanded internationally.

Overall, the mission of all enterprises aimed to improve the health and wellbeing of individuals and communities through targeted actions, services or products. Some enterprises had specific food and nutrition-based missions: addressing availability or provision of nutritious food [21, 26–29, 37, 39, 41, 44], provision of clean water [17], improving food security [19, 30, 33, 35–38, 42], accessibility of food [23, 24, 31, 32, 39, 40], affordability of food [19, 27, 29, 37, 39] and reducing food wastage [31, 42]. Other enterprises had a broader focus on improving undernutrition [22], meeting micronutrient needs [18, 25], and improving well-being [20, 43]. Some enterprises aimed to create resilient and sustainable food systems for the community of interest [24, 32, 34, 40]. Extending on their food and nutrition-based mission, some enterprises had additional aims to impact the economic development of a local area [33], provide employment opportunities [19, 28, 32, 44], tackle poverty [22, 26, 28] and support ecological sustainability [21]. See Table 1.

Enterprises had been operating for an average of 16.5 years (range: 8 months to 62 years). Out of the 21 enterprises that had verifiable information, 67% had been in existence for at least a decade. From verification, it was determined that two were no longer operating in 2024, and 10 enterprises operational status was not able to be verified.

There were eight different business models that were identified with some applying a combination of models (see Tables 2 and 3). The identified business models were: cooperatives (fee-based membership organisation); employment (provides employment opportunities for a target group); targeted customer service (charges customer directly for service or product); beneficiary service (offers social services for a small fee to low-income customers); market intermediary (the social enterprise markets or sells a client’s service or product to the buyer); social partnership (serve as brokers, connecting a client to a buyer); trading business (service or product is sold to fund an internal social program); and social business (funds external social programs by selling a service or product) (refer to Table 3 for the main features of each model).

The most common models were targeted customer service (*n* = 12), cooperative (*n* = 7) and service social business (*n* = 5). Interestingly, all models except for the employment model, had average durations of at least 10 years (see Table 3). The social cooperative model had the greatest average duration of 29 years, followed by the market intermediary model with 18 years and social partnership and social business models with 15 years of duration.

Although all of the included social enterprises had to have their own revenue stream, almost all had received external funding to either initiate their venture, create employment opportunities, or to fund the development of infrastructure or technologies. Funding sources varied and included community members; government, the food industry; non-government organisations or philanthropic organisations (including private donors); venture capitalists (business professionals who provide capital to start-ups from an investment fund in exchange for an equity stake); angel investors (high net worth individuals who invest their own money into start-ups); or a sporting team.

Some social enterprises had diverse revenue streams, and this appeared to be linked with longer-term sustainability and were more likely to have a cooperative model of business operation. For instance, the longest operational social enterprise was Norway House Fisherman’s Co-op (62 years), which invested their yearly profit into business diversification, including operating a local lumber business, a convenience store, a petrol station and a franchise that sold value-added fish products [33]. As another example, Urban Roots, although primarily focused on urban agriculture and the sale of fresh produce, charged a consultancy fee to some larger organisations with which it worked [32]. Grameen Danone Foods Ltd apply different marketing strategies and profit ratios for their yoghurt products based on whether they are selling to low-income or high-income markets [25]. 

## Discussion

This review sought to explore what food and nutrition-based social enterprise models exist that have a mission to improve health or nutrition-based outcomes. It aimed to understand their operational business models, along with other factors that contributed to their financial and operational sustainability.

There were 8 different operational models, which all except for the employment model, had endured for at least 10 years. This emphasises that the social enterprises captured are largely self-sustaining and capable of meeting the health and nutrition needs of communities. It should also be noted that two social enterprises were no-longer operating in 2024, and a further 10 were not verified as operational because information about the projects were no longer publicly available.

Variance was evident across the social enterprises' operational structure, financial structure, their mission alignment and use of resources. However, the social cooperative model clearly had the longest average duration of 29 years. This model relies on active community engagement with members having a strong financial investment, which may include an annual membership renewal fee. The social cooperative model requires ‘community ownership’, consistent with studies which describe social enterprises as community-based action that operate at a grass roots level, and are strategically aligned to the immediate social needs of a community [20, 50]. Bottom-up, community-led approaches improve the capacity of local members, which can lead to the development of individual and community-based assets that can improve health, increase social capital, build coherence and ultimately transform local food systems [51, 52]. The social cooperative model has been applied in communities with disadvantaged, low-income populations and communities of colour, and challenges existing notions of power relations to work towards food sovereignty [20, 53]. Nevertheless, entrepreneurial activities that engage private and government stakeholders remain important, since these stakeholder offer alternative revenue streams and essential capital and scale-up support [52]. The issue of social enterprises being set up and led by entrepreneurs can lead to an over reliance on individual charismatic leaders which obscure systemic barriers and with governance issues [54]. Strong independent governance processes and or differentiated leadership models may serve to support longevity [55]. 

Building a resilient business model, relies on its management or leaders having financial acumen alongside a strong financial structure and performance measures to monitor financial viability and ensure accountability [55]. This review reported on long-term social enterprises that had diverse revenue streams which contributed to their long-term sustainability and scale-up. Leveraging diverse revenue streams mitigates the risk associated with dependency on a single funding source and allowed enterprise impacts to reach low-resourced communities. However, it is important to note that almost all of the enterprises captured received external funding, either for start-up capital or periodically to sustain activities. These findings are supported by Spiess-Knafl and Jansen (2014), who describe social enterprises typically using internal financing to cover repeating operational expenses, and external financing (investors will have different expectations on financial return) to achieve scale-up or diversification [56]. Investment and support from government, philanthropic and private organisations are essential in ensuring growth, reach of the social enterprise to beneficiaries and targeted revenue-generating customers, and sustained social impact within the local context [35]. As social enterprises have become important partners in the delivery of key public services such as healthcare and health promotion, government and procedures need to develop policies that facilitate establishment of social enterprise structures and support them through the critical establishment phase, and undertake to work with entrepreneurs to manage the conflicts and trade-offs between profit-generating and non-profiting generating activities that are often problematic to balance [57]. 

Learnings from unsuccessful social enterprise models are also important. The Food Justice Truck and Sweet Water Organics were unsuccessful in sustaining their enterprise past five years. Although the Food Justice Truck had significant financial input and provided supplemental, but highly valued sources of fresh produce to asylum seekers, it was reported that the model was not sufficiently scalable or robust to meet the diverse needs of asylum seekers access to food over a wide geographical area [37]. It was reported that power imbalances were unintentionally established through prices not being displayed and some product lines not being openly available which made some customers of the Justice Truck uncomfortable [37]. Contrastingly, Sweet Water Organics struggled to manage the resource intensity of its aquaponics and could not sustain its ‘for profit’ model or its expensive adaptations [19]. It could not ultimately achieve financial independence from loans [19]. It is therefore vital to consider an enterprise’s business model, scalability and potential for financial independence from the outset. The issues of enterprises setting up without consideration of stakeholder misalignment and loss of community accountability have been described by Eiselein & Dentchev (2022) [58]. 

Another key factor which can influence the operation and sustainability of a social enterprise is having the capacity to adapt to external influences and changes. Governance structures, operations and resource distribution are often influenced by the broader economic environment. Due to social enterprises being intimately connected to civil society and the economy, models of operation and governance are shaped by political influences, the state of the economy and culture [59]. It is important to consider that social enterprises may shift responsibility from the governments to the private or not for profit sector. For example in Australia, there is evidence to suggest a resistance to food-based social enterprises for food relief [35], which is exacerbated by the ‘Good Samaritan’ legislation, acting as a barrier to establishing community supermarkets. In contrast, the United Kingdom and Canda (British Columbia) have legislative environments that allow for the sale of donated food to consumers [60]. 

In summary, the success and sustainability of the social enterprises captured were influenced by several factors. First, there was an array of business models identified that were specific to the needs of their beneficiaries and local community, supported by available resources, whilst considering the planned reach. However, when considering long-term sustainability, the social cooperative, market intermediary, social partnership and social business models were shown to have the greatest longevity. These social enterprise models rely on high level of community ownership (including effective governance and planning) and ultimately created a local food system which was more resilient to external political factors and legislative change. Finally, the success of social enterprises can be attributed to leveraging a business model that aligns with the social problem and balances “mission and money” by meeting their social mission, whilst generating diverse revenue streams used to sustain their activities with the desired social impact.

### Strengths and limitations

A key methodological strength of this review was thebreadth of searches across multiple databases embracing different disciplines, including health and business. Data that was captured in the peer-reviewed literature was cross-checked with grey literature, and verified via web searches, to better understand whether the social enterprises were existing to date.

Grey literature which may have included reports on social enterprises that can capture information on social return of investment and insights to business operations were not included. Future reviews could include program evaluations, internal reports, and policy briefs, which may offer insights into social return on investment and a more comprehensive account of the sector’s landscape. The articles from which data was abstracted ranged in academic rigour, from narrative case studies (being the majority) to more detailed mixed-methods studies with only one reporting a formal experimental evaluation of the social enterprise. This varied literature quality suggests that new enterprises are left without academic evaluations of effectiveness or impact, and therefore, there is likely to be several existing food or nutrition-based social enterprises that may not have been captured in this review.

### Implications for practice and research

This scoping review highlighted the need for more robust peer-reviewed literature with methodological strength to evaluate the impacts and process of implementation for food and nutrition based social enterprises, including their business model. A standardised typology or framework would assist in both conducting evaluations and synthesising the literature to enable more direct comparison. Future research should adopt robust, mixed-methods or experimental designs to evaluate the effectiveness, scalability, and long-term sustainability of social enterprise models. Due to the novel and topical nature of social enterprises and their potential to meet social and economic needs, there are academic opportunities to evaluate such ventures and disseminate findings for advocacy efforts. Regulatory environments that facilitate the development of place-based food and nutrition social enterprises should be prioritised, encouraging community ownership by applying co-design methodologies to ensure cultural acceptability and participatory governance. This underscores the need for policies that can support the development and implementation of the social enterprise sector within an effective regulatory environment, whilst responding to the needs of specific communities [35]. 

## Conclusion

This review sought to scope the existing literature to explore what food and nutrition-based social enterprise models exist and the characteristics pertaining to their operation. Some social enterprise business models offer promising solutions to delivering on, and sustaining food and nutrition outcomes within communities, whilst generating profit. However, for their full potential to be realised, rigorous evaluation methods are warranted to share and understand barriers and enablers to success. Longitudinal studies are necessary to monitor the development of social enterprise models over time, particularly their responsiveness to political, economic, and environmental changes. Comparative research across different jurisdictions and socio-cultural contexts would further deepen our understanding of effective strategies within specific environments. Additionally, community-led approaches should be encouraged from the beginning, especially when top-down support is inconsistent. This approach allows sufficient time for income streams to be developed, and should be facilitated by a supportive regulatory framework.

## Supplementary Information

Supplementary Material 1.

## Data Availability

Data sharing is not applicable to this article as no datasets were generated or analysed during the current study.
