# Supplementary material for: Mission and money: A scoping review of social enterprise business models as a solution to sustaining health-promoting food and nutrition-based initiatives
Source: BMC Public Health. 2025 Oct 31;25:3692. doi: 10.1186/s12889-025-24891-7 (PMC12577109; doi:10.1186/s12889-025-24891-7)
Supplement: Supplementary file 1 — Supplementary Material 1. [file 12889_2025_24891_MOESM1_ESM.docx]

**Supplementary Material 1:** Search terms for academic databases and number of results obtained

| **Database** | **Detailed Search Terms/Strategy** | **Number of Results Obtained** |
| --- | --- | --- |
| PubMed | ("Social enterprise*"[Title/Abstract] OR "Social business*"[Title/Abstract] OR "Social venture*"[Title/Abstract] OR "microenterprise*"[Title/Abstract] OR "social entrepreneur*"[Title/Abstract] OR “mission drive*”[Title/Abstract]) AND (Food*[Title/Abstract] OR nutrition*[Title/Abstract] OR “Diet, Food, and Nutrition”[Mesh] OR “food assistance”[Mesh] OR "food supply"[Mesh] OR diet*[Title/Abstract] OR nutrient* [Title/Abstract]) | 60 |
| Web of Science | ((TI="Social enterprise*" OR AB="Social enterprise*") OR (TI="Social business*" OR AB="Social business*") OR (TI="Social venture*" OR AB="Social venture*") OR (TI=microenterprise* OR AB=microenterprise*) OR (TI="social entrepreneur*" OR AB="social entrepreneur*") OR (TI="mission drive*" OR AB="mission drive*")) AND ((TI=Food* OR AB=Food*) OR (TI=nutrition* OR AB=nutrition*) OR (TI="food assistance" OR AB=“food assistance”) OR (TI="food supply" OR AB=“food supply”) OR (TI=diet* OR AB=diet*) OR (TI=nutrient* OR AB=nutrient*)) | 240 |
| Embase | ('Social enterprise*':ti,ab OR 'Social business*':ti,ab OR 'Social venture*':ti,ab OR microenterprise*:ti,ab OR 'social entrepreneur*':ti,ab OR 'mission drive*':ti,ab) AND (Food*:ti,ab OR nutrition*:ti,ab OR nutrition/exp OR 'food assistance'/exp OR 'catering service'/exp OR diet*:ti,ab OR nutrient*:ti,ab) | 92 |
| Cinahl | ((TI "Social enterprise*" OR AB "Social enterprise*") OR (TI "Social business*" OR AB "Social business*") OR (TI "Social venture*" OR AB "Social venture*") OR (TI microenterprise* OR AB microenterprise*) OR (TI "social entrepreneur*" OR AB "social entrepreneur*") OR (TI "mission drive*" OR AB "mission drive*")) AND ((TI Food* OR AB Food*) OR (TI nutrition* OR AB nutrition*) OR (MH "Nutrition+") OR (MH "Food Assistance+") OR (MH "Food Supply+") OR (TI diet* OR AB diet*) OR (TI nutrient* OR AB nutrient*)) | 25 |
| Social Science Database | (MAINSUBJECT.EXACT("Social entrepreneurship") OR TI,AB("Social enterprise*") OR TI,AB("Social business*") OR TI,AB("Social venture*") OR TI,AB(microenterprise*) OR TI,AB("social entrepreneur*") OR TI,AB("mission drive*")) AND (TI,AB(Food*) OR TI,AB(nutrition*) OR MAINSUBJECT.EXACT("Nutrition") OR MAINSUBJECT.EXACT("Food supply") OR TI,AB(diet*) OR TI,AB(nutrient*)) | 57 |
| Sociological Abstracts | (MAINSUBJECT.EXACT.EXPLODE("Social entrepreneurship") OR TI,AB("Social enterprise*") OR TI,AB("Social business*") OR TI,AB("Social venture*") OR TI,AB(microenterprise*) OR TI,AB("social entrepreneur*") OR TI,AB("mission drive*")) AND (TI,AB(Food*) OR TI,AB(nutrition*) OR MAINSUBJECT.EXACT.EXPLODE("Nutrition") OR TI,AB(diet*) OR TI,AB(nutrient*)) | 49 |
| Business Source Complete | ((DE "SOCIAL enterprises") OR (DE "SOCIAL entrepreneurship") OR (DE "SOCIAL enterprises") OR (TI "Social enterprise*" OR AB "Social enterprise*") OR (TI "Social business*" OR AB "Social business*") OR (TI "Social venture*" OR AB "Social venture*") OR (TI microenterprise* OR AB microenterprise*) OR (TI "social entrepreneur*" OR AB "social entrepreneur*") OR (TI "mission drive*" OR AB "mission drive*")) AND ((TI Food* OR AB Food*) OR (TI nutrition* OR AB nutrition*) OR (DE "COMMUNITY food services") OR (DE “FOOD supply”) OR (TI diet* OR AB diet*) OR (TI nutrient* OR AB nutrient*)) | 208 |
| ABI/Inform Collection | (MAINSUBJECT.EXACT("Entrepreneurship") OR TI,AB("Social enterprise*") OR TI,AB("Social business*") OR TI,AB("Social venture*") OR TI,AB(microenterprise*) OR TI,AB("social entrepreneur*") OR TI,AB("mission drive*")) AND (TI,AB(Food*) OR TI,AB(nutrition*) OR MAINSUBJECT.EXACT("Nutrition") OR MAINSUBJECT.EXACT("Food supply") OR TI,AB(diet*) OR TI,AB(nutrient*)) | 1771 |
